# Treatment Plan Technique and Quality for Single-Isocenter Stereotactic Ablative Radiotherapy of Multiple Lung Lesions with Volumetric-Modulated Arc Therapy or Intensity-Modulated Radiosurgery

**DOI:** 10.3389/fonc.2015.00213

**Published:** 2015-10-06

**Authors:** Kimmen Quan, Karen M. Xu, Ron Lalonde, Zachary D. Horne, Mark E. Bernard, Chuck McCoy, David A. Clump, Steven A. Burton, Dwight E. Heron

**Affiliations:** ^1^Department of Radiation Oncology, University of Pittsburgh Cancer Institute, Pittsburgh, PA, USA

**Keywords:** single-isocenter, VMAT, IMRs, multi-lesion lung cancer, plan technique and quality

## Abstract

The aim of this study is to provide a practical approach to the planning technique and evaluation of plan quality for the multi-lesion, single-isocenter stereotactic ablative radiotherapy (SABR) of the lung. Eleven patients with two or more lung lesions underwent single-isocenter volumetric-modulated arc therapy (VMAT) radiosurgery or IMRS. All plans were normalized to the target maximum dose. For each plan, all targets were treated to the same dose. Plan conformity and dose gradient were maximized with dose-control tuning structures surrounding targets. For comparison, multi-isocenter plans were retrospectively created for four patients. Conformity index (CI), homogeneity index (HI), gradient index (GI), and gradient distance (GD) were calculated for each plan. V5, V10, and V20 of the lung and organs at risk (OARs) were collected. Treatment time and total monitor units (MUs) were also recorded. One patient had four lesions and the remainder had two lesions. Six patients received VMAT and five patients received intensity-modulated radiosurgery (IMRS). For those treated with VMAT, two patients received 3-arc VMAT and four received 2-arc VMAT. For those treated with IMRS, two patients were treated with 10 and 11 beams, respectively, and the rest received 12 beams. Prescription doses ranged from 30 to 54 Gy in three to five fractions. The median prescribed isodose line was 84% (range: 80–86%). The median maximum dose was 57.1 Gy (range: 35.7–65.1 Gy). The mean combined PTV was 49.57 cm^3^ (range: 14.90–87.38 cm^3^). For single-isocenter plans, the median CI was 1.15 (range: 0.97–1.53). The median HI was 1.19 (range: 1.16–1.28). The median GI was 4.60 (range: 4.16–7.37). The median maximum radiation dose (D_max_) to total lung was 55.6 Gy (range: 35.7–62.0 Gy). The median mean radiation dose to the lung (D_mean_) was 4.2 Gy (range: 1.1–9.3 Gy). The median lung V5 was 18.7% (range: 3.8–41.3%). There was no significant difference in CI, HI, GI, GD, V5, V10, and V20 (lung, heart, trachea, esophagus, and spinal cord) between single-isocenter and multi-isocenter plans. This multi-lesion, single-isocenter lung SABR planning technique demonstrated excellent plan quality and clinical efficiency and is recommended for radiosurgical treatment of two or more lung targets for well-suited patients.

## Introduction

Stereotactic ablative radiotherapy (SABR) has been shown to be effective in treating inoperable patients with primary or metastatic lung lesions (1, 2). Traditionally, the delivery systems for SABR include multiple coplanar and/or non-coplanar beams and helical tomotherapy (3, 4). With recent technological advances, volumetric-modulated arc therapy (VMAT) and intensity-modulated radiosurgery (IMRS) have been established as treatment techniques for delivering SABR to the lung (5–14). They provide optimal dose distributions and precise targeting with excellent treatment delivery efficiency. Recent studies have shown that single-isocenter VMAT technique is feasible and provides highly conformal dose distributions, good plan quality, and short treatment delivery times compared with conventional multiple-isocenter technique for multi-target intracranial radiosurgery (15, 16). In 2014, Zhang et al. (17) reported nine non-small cell lung carcinoma patients treated with single-isocenter coplanar and non-coplanar intensity-modulated radiation therapy (IMRT) and Tomotherapy (Version 4.2, Madison, WI). Liu et al. (18) reported the use of single-isocenter multisegment dynamic conformal arc (SiMs-arc), full-arc VMAT, and partial-arc VMAT techniques for lung SABR in five patients. Similarly, we hypothesize that SABR delivered with single-isocenter VMAT (RapidArc, Varian Medical Systems, Palo Alto, CA, USA) or IMRS (TrueBeam STx linear accelerator, Varian Medical Systems, Palo, Alto, CA, USA) is a feasible treatment technique for multi-target primary or oligometastatic lung nodules. This technique could potentially save treatment time and make treatment planning more convenient.

Our institution has adopted single-isocenter VMAT or IMRS technique for delivering SABR to multiple lung lesions. In this article, we report our practical and systematic treatment planning techniques and evaluate planning qualities and dosimetric parameters of single-isocenter VMAT or IMRS techniques versus multi-isocenter VMAT techniques for a cohort of patients diagnosed with two or more primary or oligometastatic pulmonary lesions.

## Materials and Methods

### Patient characteristics

From January 2011 to September 2014, 11 patients with multiple primary or oligometastatic lung lesions were treated with a frameless single-isocenter VMAT or IMRS radiosurgery technique. Four patients were males and seven patients were females. The median age was 73.1 years old (range: 51–87 years old). One patient had four lesions and the other 10 patients had two lesions. Eight patients had lesions on the same lobe and three patients had lesions on two adjacent lobes. Seven patients had primary lung cancers while the remaining patients had oligometastatic disease; one had primary colorectal cancer and two had primary head and neck cancer. One patient had disease of unknown histology. Seven patients had adenocarcinoma while three patients had squamous cell carcinoma. All patients had lesions in relative proximity to each other, which either had minimal motion or moved in the same direction during breathing.

The decision to treat with a single isocenter was based on a number of factors: the overall volume of the lesions, the distance between them (<7 cm), and whether the motion of the two lesions (as characterized in a 4-dimensional computed tomography (4D-CT) scan) was similar. Single-isocenter planning was not attempted for targets with separation >7 cm since rotational setup errors may lead to position offsets in lesions, the further away they are from the isocenter.

### Treatment planning techniques

Patients were immobilized with the Elekta Body Fix (Elekta Oncology Systems, Crawley, UK) coupled with a vacuum cover sheet to immobilize the patient in the cushion. Patients then underwent 4D-CT scan using GE LightSpeed 16 scanner (GE Healthcare, Waukesha, WI) under audio coaching. The respiratory cycle was set as 3–5 s based on an individualized natural breathing pattern. For each patient, 10 sets of phase-sorted CT images with a slice thickness of 2.5 mm were acquired using GE advantage 4D workstation (GE Healthcare) and imported into Varian Eclipse treatment planning system (Varian Medical Systems, Palo Alto, CA, USA) for further processing. Gross tumor volumes (GTVs) were contoured on CT images at phase 50%, which represents the end of expiration. Afterwards, a customized margin was added to generate the GITV. An isotropic direct expansion of 5 mm was used to generate the PTV. Increasing the distance between targets potentially decreases the plan quality. Thus, our single-isocenter technique was only used when targets were relatively close together. However, we did not apply any specific cutoff threshold for the distance between targets. In addition, when utilizing the single-isocenter technique, it was required that targets were moving approximately in synchronization with each other.

Critical structures were also delineated. Dose-control tuning structures were then created as avoidance structures to be used in the computer optimization of dose distribution. These structures were intended to aid in optimizing two dose level areas: high (~prescription dose) and low (~50% of the prescription dose). Tuning structures were created as concentric volumetric rings. The inner control had an inner edge at the border of the PTV and outer edge of 7 mm from the PTV. The outer control had an inner edge of 7 mm from the PTV and outer edge of 1.5 cm away from the PTV. Since most patients had only two lesions, the isocenter was placed at the geometric midpoint between the lesions. Two to three coplanar 180° half arcs were used for VMAT and 10 to 12 coplanar beams were used for IMRS with the goal of creating a plan that accomplished optimal dose distributions with as few arcs and beams as possible. All plans used high-intensity flattening filter-free (FFF) mode at a dose rate of 1400 monitor units (MUs) per minute.

Several dose prescriptions and fractionation schemes were used based on protocol designation or physician preference, but treatment planning techniques for all patients followed the above methodology. All plans were normalized to deliver 100% of the prescription dose to the target maximum.

During treatment delivery, we confirmed that tumor motion was similar to that of the 4D-CT by doing a fluoroscopy prior to the treatment. Then we utilized the cone-beam CT (CBCT) and aligned it to the planning CT. If significant rotational errors were observed, the patient setup was adjusted. If the images still did not align properly after adjustments, we would not deliver the treatment. No re-imaging of the patient was done mid-treatment since it was generally faster just to complete the treatment as most of the plans were delivered with VMAT.

The SABR treatments of four patients were also retrospectively re-planned with multi-isocenter VMAT approach. Dosimetric parameters (PTV volume, maximum radiation dose, mean radiation dose, V5, V10, and V20) of organs at risk (OARs), including lung, heart, trachea, esophagus, and spinal cord for both single-isocenter and multi-isocenter plans were recorded for comparison.

### Plan evaluation

The dose calculation algorithm utilized to calculate the plans was AAA, version 11. Ideally, for all treatment plans, the PTV is covered by 100% of the prescription dose. However, practically, any plan with a PTV covered by more than 95% prescription dose is acceptable. Three major indices, conformity index (CI), gradient index (GI) and homogeneity index (HI), were used to evaluate the treatment plan. CI is calculated based on International Commission on Radiation Units and Measurements 62: CI = V_Rx_/V_PTV_, where V_Rx_ is the volume of the prescription dose and V_PTV_ is the volume of the PTV. A perfectly conformal plan generates a CI = 1. For less conformal plans, CI > 1 if the target volume was over-covered by the prescription volume and CI < 1 if the target volume was under-covered by the prescription volume. According to the Radiation Therapy Oncology Group (RTOG) Protocol 0915, CI <1.2 is acceptable and CI of 1.2–1.5 is minor deviation. GI is defined as GI = V_50%Rx_/V_100%Rx_, where V_50%Rx_ is the volume of the 50% prescribed isodose line and V_100%Rx_ is the volume of the 100% prescribed isodose line (19). Per RTOG protocol 0915, GI has to be smaller than 3–6, depending on the PTV volume. HI is defined as HI = *D*_max_/*D*_Rx_, where *D*_max_ is the maximum dose and *D*_Rx_ is the prescription dose and is used to evaluate the uniformity of dose distribution within a PTV. In addition, per RTOG Protocol 0915, lung V20 has to be smaller than 10–15%. Notably, these values were all calculated based on single-lesion radiosurgery plans.

### Treatment delivery

All plans were created for a Varian TrueBeam^TM^ STx linear accelerator (Varian Medical Systems) equipped with the Varian High Definition 120 MLC, with 2.5-mm leaf width in the central 8 cm of the field and 5 mm in the outer portion of the field. FFF mode was used for all patients with a maximal dose-delivery rate of 1400 MU/min. If 4D-CT motion study showed tumor movement more than 5 mm, gating with Varian real-time position management (RPM) system was required. A gating window centering at the 50% phase of the respiratory cycle was generated so that radiation was only delivered when tumor motion is within 5 mm. Prior to each treatment, on-board imaging (OBI) was performed with CBCT scans. Appropriate shifts were made to align targets and normal structures on OBI with those on offline planning images to ensure appropriate patient positioning. Treatment position was verified by both the physicist and the radiation oncologist. The beam-on time for delivering one fraction of each single-isocenter plans was recorded. Total MUs during one fraction of SABR delivery for both single-isocenter plans and multi-isocenter re-plans were recorded for comparison.

## Results

### Treatment planning characteristics

The majority of tumors were of non-small cell lung origins. All patients were treated with curative intents for either primary lung or oligometastatic diseases. The median follow-up time since the completion of SABR treatment was 15.8 months (range: 4.2–28.8 months). Table 1 outlines details of each single-isocenter plan such as tumor locations, radiation dose, and delivery techniques. Patient 8 had four lesions treated simultaneously, while the remainder of patients had two lesions. Three patients had lesions in adjacent lobes. Based on the treating physician and physicist’s discretions, six patients were treated with VMAT plans and five were treated with IMRS. For those treated with VMAT, two patients received 3-arc VMAT and four received 2-arc VMAT. For those treated with IMRS, one received 11 beams while one received 10 beams and the reminder received 12 beams. Prescription doses ranged from 30 to 54 Gy in three to five fractions. Additionally, we reported on inter-lesion distances for each patient in two forms. The minimum inter-lesion distance is the shortest linear distance from tumor-edge to tumor-edge in either the axial, sagittal, or coronal planes. Three patients had lesions that were non-coplanar. The 3-dimensional distance was calculated using the *x*, *y*, and *z* coordinates of lesion centers as determined by Eclipse. For Patient 8, the longest 3-D distance is reported among the four lesions.

**Table 1:** **Planning details for single-isocenter plans**.

Patient	Number of targets	Dose (Gy)	Fractions	Isodose line^f^ (%)	Lesion location	Minimum inter-lesion distance (cm)^a^	Three-dimensional distance (cm)^b^	Technique	Number of arcs or beams
1	2	48	4	86	RLL	0.43	2.32	IMRS	10
2	2	48	4	85	RUL	0.3	2.65	VMAT	2
3	2	30	3	84	RUL	0.95	3.03	IMRS	11
4	2	48	4	84	RLL	1.47	3.43	VMAT	3
5	2	48	4	80	RUL	–	3.73	VMAT	2
6	2	40	5	85	RLL	0.81	4.06	IMRS	12
7	2	54	3	83	RLL^c^	2.98	5.17	VMAT	3
8	2	48	4	80	LUL,LLL	2.97	5.63	IMRS	12
9	4	48	4	82	LULx3 LLLx1	0.21	5.74^d^	IMRS	12
10	2	54	3	85	RUL,RLL	–	6.94	VMAT	2
11	2	48	4	86	LUL	–	7.21	VMAT	2
Mean		46.7		84		1.27	4.42		
Median	2	48	4	84		0.88	3.90		2/12
SD^e^		6.6		2		1.13	1.74		
Range	2–4	30–54	3–5	80–86		0.21–2.98	2.32–7.21		

*^a^The shortest one-dimensional distance between lesion edges in an axial, sagittal, or coronal plane*.

*^b^The three-dimensional distance between lesion centers*.

*^c^RLL, right lower lobe; RUL, right upper lobe; LLL, left lower lobe; LUL, left upper lobe*.

*^d^Represents the longest inter-lesion distance among all four lesions*.

*^e^SD*.

*^f^This is the isodose line that received prescription dose*.

**Figure 1:**
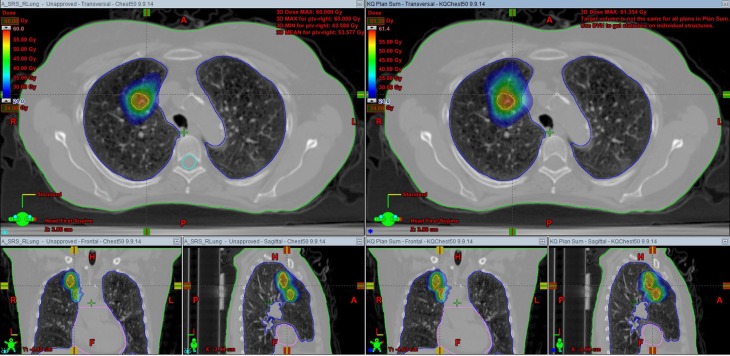
**CT scans of the target, with a colorwash display of the dose (24 Gy and above) for a 48 Gy plan, showing the smaller spread of the intermediate dose distribution in the single-isocenter plan**. Left, single-isocenter plan; right, multi-isocenter plan.

Table 2 outlines details of each multi-isocenter plan. Patients 1, 7, 9, and 11 were retrospectively re-planned with multi-isocenter VMAT techniques. Patient 1 received 3-arc VMAT and the others received 2-arc VMAT. The SABR dose to each lesion ranged from 29.252 to 54 Gy, delivered in three to four fractions. Figure 1 showed both single-isocenter and multi-isocenter treatment plans for the same target. Noticeably, the single-isocenter plan had a smaller spread of the intermediate dose distribution.

**Table 2:** **Planning details for multi-isocenter plans**.

Patient	Number of targets	Target	Dose (Gy)	Fractions	Isodose line (%)	Distance between isocenters (cm)	Techniques	Number of arcs or beams
5	2	RUL, lesion 1	48	4	82		VMAT	2
		RUL, lesion 2	48	4	80	3.7	VMAT	2
7	2	RLL, lesion 1	29.252	3	80		VMAT	3
		RLL, lesion 2	54	3	83	5.2	VMAT	3
10	2	RLL, lesion 1	54	3	86		VMAT	2
		RUL, lesion 2	54	3	87	6.9	VMAT	2
11	2	LUL, lesion 1	48	4	83		VMAT	2
		LUL, lesion 2	48	4	80	7.2	VMAT	2

*RUL, right upper lobe; RLL, right lower lobe; LUL, left upper lobe; RUL, right upper lobe*.

### Dosimetric parameters of OAR

A comparison of dose volume histogram (DVH) data for single-isocenter and multi-isocenter plans (patients 5, 7, 10, and 11) was listed in Table 3. Critical OARs included lung, heart, trachea, esophagus, and spinal cord. DVH parameters reported included irradiated organ volume, maximum radiation dose (*D*_max_), mean radiation dose (*D*_mean_), V5, V10, and V20.

**Table 3:** **Comparison of dose volume histogram (DVH) parameters for single-isocenter versus multi-isocenter treatment plans**.

Organ	Plan type	Volume (cm^3^)	*D*_max_ (Gy)	*D*_mean_ (Gy)	V5 (%)	V10 (%)	V20 (%)
Lung	Single-isocenter	3233.6 (2164.5–4633.7)^a^	60.4 (55.1–62)	4 (3.3–6.1)	18.25 (16.4–28.7)	11 (9.9–19.1)	5.1 (3.4–8.4)
	Multi-isocenter	3233.6 (2164.5–4633.7)	61.05 (59.5–66.2)	4.05 (3.3–6.5)	18.95 (17.1–28.6)	11.8 (9.4–20.8)	5.05 (3.1–9.6)
Heart	Single-isocenter	862.05 (618.1–1161.9)	13.55 (2.4–18.6)	1.45 (0.2–3.9)	7.2 (0–23.2)	0.42 (0–2.5)	0 (0)
	Multi-isocenter	862.15 (618.1–1161.9)	12.15 (2.5–21.1)	0.95 (0.2–3.6)	3.15 (0–23)	0.4 (0–4)	0 (0–0.008)
Trachea	Single-isocenter	20.15 (11.9–23.6)	17.8 (10.4–23.6)	4.9 (2.8–7.7)	44.25 (8.6–71.2)	10.1 (0.2–18.3)	0.15 (0–3.8)
	Multi-isocenter	20.15 (11.9–23.7)	18.7 (13.5–24.7)	5.4 (1.9–6.6)	52.05 (10.6–68.4)	10.85 (4.8–15.6)	0 (0–0.6)
Esophagus	Single-isocenter	35.45 (15.6–104.4)	19.25 (5.4–40.3)	6.05 (2.2–14.7)	34.4 (0.5–76.3)	7.6 (0–21)	0 (0–0.06)
	Multi-isocenter	35.45 (15.6–104.4)	9.4 (6.8–15)	2.6 (2.5–4.2)	23 (13.8–32.5)	0.1 (0–2.1)	0 (0)
Spinal cord	Single-isocenter	36.35 (18.6–60.1)	8.25 (5.4–10.8)	2.05 (1.8–7.5)	16.45 (0.4–97.5)	0 (0–0.9)	0 (0)
	Multi-isocenter	36.35 (18.6–60.1)	7.8 (5.9–9.9)	1.9 (1.6–5.4)	9.5 (1.6–61.5)	0 (0)	0 (0)

*^a^The data are presented as median (range)*.

### Lung

**Figure 2:**
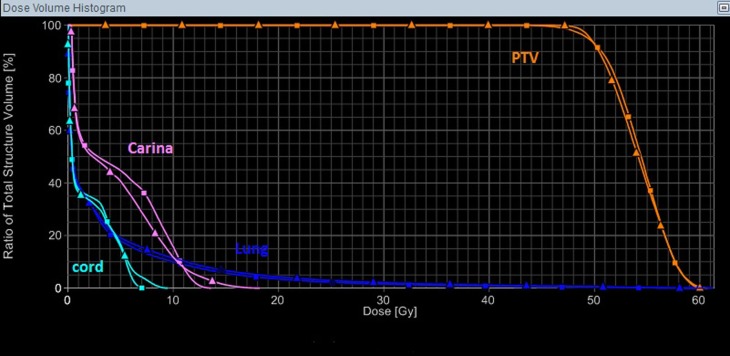
**A DVH comparison for the target and a few organs in the same plan as in Figure 1, with the triangle symbols representing the multi-isocenter plan, and the square symbols representing the single-isocenter plan**. In this case, the single-isocenter plan was overall slightly better than the multi-isocenter plan, and would take substantially less time to deliver.

V20 was smaller than 10.5% for all patients except Patient 8 with a V20 of 20.45%. Note that this patient is the only one having four lung lesions with a total tumor volume of 87.38 cm^3^. Furthermore, Patient 8 had a left-sided pacemaker, which required an upper limit of radiation dose of 2 Gy. For those four patients with both single-isocenter and multi-isocenter plans, the dosimetric parameters for both plans were listed in Table S1 in Supplementary Material. Figure 2 showed a dose-volume histogram (DVH) comparison for the target and a few organs in the same plan as in Figure 1.

### Heart

For those four patients with both single-isocenter and multi-isocenter plans, the dosimetric parameters for both plans were listed in Table S2 in Supplementary Material.

### Trachea

For those four patients with both single-isocenter and multi-isocenter plans, the dosimetric parameters for both plans were listed in Table S3 in Supplementary Material.

### Esophagus

For those four patients with both single-isocenter and multi-isocenter plans, the dosimetric parameters for both plans were listed in Table S4 in Supplementary Material.

### Spinal cord

For those four patients with both single-isocenter and multi-isocenter plans, the dosimetric parameters for both plans were listed in Table S5 in Supplementary Material.

### Treatment plan evaluation

We assessed each single-isocenter plan’s dose conformity for, homogeneity within, and falloff outside the combined PTV, listed in Table S6 in Supplementary Material. CI for all patients was smaller than or close to 1.5. GI for all plans except the plan for Patient 8 was approximately within the constraints corresponding to each target volume criteria provided by the RTOG Protocol 0915. The details of plan evaluation parameters for multi-isocenter plans were listed in Table S7 in Supplementary Material. Table 4 shows a direct comparison of single-isocenter and multi-isocenter plan evaluation parameters.

**Table 4:** **Comparison of plan evaluation parameters for single-isocenter versus multi-isocenter treatment plans**.

	Target	Combined PTV (cm^3^)	Conformity index	Homogeneity index	Gradient index	Gradient distance (cm)
5	Single-isocenter	14.9	1.19	1.25	5.72	1.25
	RUL, lesion 1	5.6	1.22	0.94	6.98	1.14
	RUL, lesion 2	9.3	1.25	1.17	6.49	0.97
7	Single-isocenter	46	1.21	1.2	4.16	1.41
	RLL, lesion 1	10.9	1.13	1.25	4.34	1.17
	RLL, lesion 2	35.1	1.22	1.20	4.64	0.96
10	Single-isocenter	15.21	1.53	1.18	6.10	1.45
	RLL, lesion 1	7.3	1.14	1.16	5.34	0.93
	RUL, lesion 2	8	1.14	1.15	5.60	0.99
11	Single-isocenter	75.18	0.97	1.16	4.41	1.64
	LUL, lesion 1	40.4	2.84	1.20	3.76	1.21
	LUL, lesion 2	34.7	1.27	1.25	4.12	1.29

### Total monitor units and treatment time

For single-isocenter plans, the median treatment time for one fraction of SABR delivery was 6.4 min (range: 2.7–19 min) for those treated with VMAT and was 19.1 min (range: 8.3–40.9 min) for those treated with IMRS. The median total MUs for one fraction of SABR delivery was 4423 MUs (range: 3106–6049 MUs) for those treated with VMAT and was 5831 MUs (range: 4537–11449 MUs) for those treated with IMRS. For multi-isocenter plans, the median total MUs was 8116 MUs (range: 6016–9950 MUs). The details were listed in Table 5.

**Table 5:** **Treatment time and total monitor units for single-isocenter and multi-isocenter plans**.

Patient	Single-isocenter			Multi-isocenter	
	Treatment modality	Time (min)	Total MUs	Treatment modality	Total MUs
1	IMRS	16.22	4537		
2	VMAT	7.09	4957		
3	IMRS	8.34	5831		
4	VMAT	3.17	3691		
5	VMAT	2.66	3106	VMAT	7699
6	IMRS	40.88	11449		
7	VMAT	18.94	5304	VMAT	9950
8	IMRS	19.11	5350		
9	IMRS	23.06	8071		
10	VMAT	5.61	6049	VMAT	8533
11	VMAT	13.89	3889	VMAT	6016

## Discussion

This multi-lesion, single-isocenter VMAT or IMRS SABR planning technique produces plans of high quality and excellent clinical efficiency, especially when combined with a high-intensity FFF beam with a maximal dose-delivery rate of 1400 MU/min. It is a potential replacement for multi-isocenter techniques for multiple targets in selected candidates. In this report, we presented a systematic framework for the creation of single-isocenter VMAT or IMRS plans for the treatment of multiple lung lesions.

This single-isocenter approach improves clinical efficiency significantly. For the treatment of multiple brain tumors, Clark et al. showed that the triple-arc single-isocenter VMAT plan had a delivery time of 6 min, while the triple-arc triple-isocenter VMAT plan had a delivery time of 14 min (15). Nath et al. reported a median delivery time of 21 min using the single-isocenter IMRS technique to treat five lesions, while the four-isocenter IMRS technique took about 38 min (20). From our experience, the single-isocenter VMAT or IMRS technique for the treatment of multiple lung tumors also took much less time than the multi-isocenter technique, as evidenced by our recorded treatment time. At our institution, we often use two to three 180° arcs for the VMAT technique and 10–15 beams for the IMRS technique. Each arc takes about 1.5–2 min to finish. Around 6 min is required to finish three arcs with a single-isocenter plan. With the conventional two-isocenter approach for two lung lesions, 12 min would be required to finish the treatment, not accounting for the setup time for the second isocenter. Similarly, single-isocenter approach with IMRS will also decrease the treatment time substantially. Improved clinical efficiency is seen when combining the single-isocenter setup with a linear accelerator equipped with high-intensity FFF technology delivering 1400 MU/min. Decreasing the treatment time may reduce intrafraction motion, which may allow reduced PTV margins in some patients. In addition, it is difficult for many patients with advanced lung disease to lie flat for extended periods of time. Reducing the treatment time may translate to improved patient comfort and easier radiation delivery.

Four-dimensional computed tomography has become the standard radioimaging tool for assessing lung tumor volume, position, and motion during treatment planning (21). Although it is assumed that the tumor motion captured with 4D-CT at the time of planning is representative of tumor motion during treatment, changes in respiratory patterns have been observed in patients undergoing conventionally fractionated radiotherapy (22, 23). The resulting geographic errors can compromise local control and cause unnecessary irradiation of healthy tissues, increasing the chance of radiation toxicity. Reducing the treatment time decreases the likelihood of changes in respiratory patterns from coughing or discomfort and makes geographic miss less likely (24).

In thoracic radiotherapy, radiation pneumonitis and pulmonary fibrosis are the most common and the major dose-limiting toxicities (25). Lung V20 and V5 are potential predictors of the risk for radiation-induced pneumonitis. Lung V20 should be less than 10% and lung V5 should be less than 40% (26) to minimize the likelihood of developing symptomatic pneumonitis (27, 28). Except Patient 8 (four lesions), V20 for all patients was lower than or close to 10.5% and V5 for all patients was lower than 40%. No patient developed symptomatic radiation pneumonitis after receiving treatment. Moderate dose spills outside the PTV can be correlated with a higher risk of complications. The GI is an excellent surrogate for the evaluation of dose spill. Per RTOG protocol 0915, a constraint for GI exists, depending on the PTV volume. For PTV values ranging from 13.2 to 95 cm^3^, the upper limit of GI ranges from 4.4 to 5.8. Except Patients 7 and 8, all patients had a GI within this range, which translates to minimal dose spill. CI for all patients was less than or close to 1.5. The lowest CI was 0.97. HI was within a range of 1.16–1.28, corresponding to good uniformity of dose distributions within the PTV. Gradient distance (GD) is the average distance from 100% prescribed dose to 50% prescribed dose and ranged from 1.14 to 2.69 cm among our patients. It is an indicator of how fast the radiation dose falls off and is used to evaluate the sparing of normal lung tissues. These parameters demonstrated that the single-isocenter VMAT or IMRS plans created for these patients were of excellent quality. It is worth noting that Patient 8 had a left-sided pacemaker requiring a constraint of maximum radiation dose of 2 Gy when treating his four left-sided lung lesions. This challenge made the plan more complicated than the rest, but the dosimetric parameters only exceeded the constraints slightly and no toxicity has been reported. Therefore, it is very promising that these single-isocenter SABR of the lung plans will become extremely customizable and widely applied. Although our study used Varian Eclipse treatment planning software and a Varian TrueBeam linear accelerator, the outlined treatment planning techniques and principles should be applicable to other treatment planning and delivery platforms. Our experience showed that the integration of dose-control tuning structures with the optimization goals leads to great improvement in overall plan quality and it could be implemented on other treatment planning platforms as well.

Another potential concern for single-isocenter treatment plans is leakage dose from leaves moving in between tumors, which may cause increase of the low-dose region. V5, V10, and V20 are good measures of the low-dose regions. As shown in Table 3, our single-isocenter plans had similar or even smaller V5, V10, and V20, compared with multi-isocenter plans. In Table S7 in Supplementary Material, we listed the plan evaluation parameters for multi-isocenter plans. For Patient 7, the CI for the single-isocenter plan was 1.53, higher than those for the multi-isocenter plan (CI = 1.14), but still within a reasonable range. GDs for single-isocenter plans were increased compared with those of the multi-isocenter plans, which indicated slower fall off of doses. This suggested that single-isocenter plans may cause greater radiation to normal lung tissues or adjacent OARs. However, as mentioned, GIs for all single-isocenter plans except Patients 7 and 8 were all within the constraints suggested by RTOG protocol 0915. In addition, in Tables S1–S5 in Supplementary Material, we listed volume, *D*_max_, *D*_mean_, V5, V10, and V20 for total lung, heart, trachea, esophagus, and spinal cord of both single-isocenter and multi-isocenter plans. For esophagus and spinal cord, single-isocenter plans tended to produce more radiation, but *D*_max_ values of esophagus for all plans (except Patient 11) and spinal cord for all plans were less than 30 and 26 Gy, respectively, as suggested by the RTOG protocol 0915.

Intensity-modulated radiosurgery allows for adjustment of the intensity of the radiation beam so that the tumor receives a high dose of radiation while minimizing radiation exposure to surrounding normal tissues. Therefore, it may improve the therapeutic ratio for lung cancers, increasing the beneficial effect while minimizing the toxicity. However, highly conformal therapy requires accurate target definition and assessment of target motion. Only the tissues identified as the target will receive therapeutic dose and slight geographic error may lead to significant toxicities (29). In addition, the planning and quality assurance processes are very complex and time-consuming. A recent report by Zhang et al. introduced the single-isocenter technique for treating two or three lung lesions in nine patients with IMRS or tomotherapy. However, nine static beams were used in all of their plans while the beam number in our IMRS plans was individualized, with a range of 10–12 beams. Using more beams improves plan quality and the required number of beams depends directly on the complexity of the fluence profiles that can be delivered within the physical and technical constraints of the treatment machine (30). On the other hand, VMAT has a much faster delivery time and also has the ability to achieve highly conformal dose distributions. It is considered as an alternative form of IMRS (31). A recent report by Liu et al. showed that additional intensity modulation allowed VMAT to produce dosimetrically improved plans than the SiMs-arc techniques in five patients (18). It was also noted that due to inverse planning with overlapping arcs, half-arc VMAT may produce potentially more superior treatment plans than full-arc VMAT. However, unlike our plans, the isocenter of the half-arc VMAT plans reported in Liu et al. was placed inside the PTV. In addition, all five patients mentioned in Liu et al. had two lesions on the same lobe while one patient in our report had two lesions on adjacent lobes. The choice of IMRS or VMAT depends on the specific treatment planning goals and preferences of the radiation oncologist and the physicist.

However, this single-isocenter SABR of the lung technique does have several limitations, including potential setup errors, motion errors, and rotational errors. For two lesions, the isocenter is usually placed at the midpoint of the lesions. With multiple (≥4) non-coplanar lesions, sometimes it can be difficult to determine the optimal position for the isocenter. As we mentioned previously, some variability exists in respiratory patterns between the treatment and the planning sessions. Due to the high conformity of IMRS and VMAT, even slight motion error can lead to a significant risk of geographic miss and toxicities. In addition, lesions separated by more than a few centimeters may exhibit different respiratory motions, requiring careful definition of PTV margins for each. Dose distribution for this technique is also very sensitive to rotational errors, especially for small lesions and those distant from the isocenter. Therefore, great care must be taken to ensure accurate imaging guidance and patient positioning for this single-isocenter multi-target technique. Increasing the distance between targets potentially decreases the plan quality. Thus, our single-isocenter technique was only utilized when targets were relatively close together. However, we did not apply any cutoff threshold for the distance. In addition, before the treatment planning, an evaluation of each target’s motion was made with the 4D-CT to determine whether targets were moving in synchronization with each other, an ideal scenario for our single-isocenter technique.

## Conclusion

Our report is the largest series so far to introduce the single-isocenter SABR of the lung with VMAT or IMRS. In addition, we report the very first single-isocenter IMRS plan for four lung lesions on two adjacent lobes. Compared to previous studies, our treatment plans use better techniques, including individualizing and increasing the beam number in IMRS and applying half-arc VMAT with the isocenter placed outside the PTV. We also compared plan evaluation parameters and dosimetric parameters between single-isocenter and multi-isocenter plans. The results of our report showed that this technique produces plans of favorable CI, GI and HI values, which are surrogates of high clinical quality. We have introduced a practical and systematic framework for radiation oncologists, physicists, and dosimetrists to utilize when creating single-isocenter VMAT or IMRS lung radiosurgery plans. The excellent plan quality and clinical efficiency makes single-isocenter approach a potential replacement for the currently adopted multi-isocenter approach.

## Ethics Approval

This study was carried out in accordance with the recommendations of the Institutional Review Board (PRO 13020306) with written informed consent from the respective subjects.

## Conflict of Interest Statement

The authors declare that the research was conducted in the absence of any commercial or financial relationships that could be construed as a potential conflict of interest.

## Supplementary Material

The Supplementary Material for this article can be found online at http://journal.frontiersin.org/article/10.3389/fonc.2015.00213
