# Supplementary material for: Treatment Plan Technique and Quality for Single-Isocenter Stereotactic Ablative Radiotherapy of Multiple Lung Lesions with Volumetric-Modulated Arc Therapy or Intensity-Modulated Radiosurgery
Source: Front Oncol. 2015 Oct 6;5:213. doi: 10.3389/fonc.2015.00213 (PMC4594030; doi:10.3389/fonc.2015.00213)
Supplement: Supplementary file 1 [file DataSheet_1.PDF]

Table S1. Single-isocenter versus multi-isocenter plan dosimetric parameters for total lung

Patient	Plan type	Volume (cm^3)	D_{max} (Gy)	D_{mean} (Gy)	V5 (%)	V10 (%)	V20 (%)
5	Single-isocenter	3696.9	59.5	3.3	17.8	9.9	3.4
	Multi-isocenter	3696.9	61.9	3.7	19.1	11.4	4.3
7	Single-isocenter	2164.5	62.0	4.2	16.4	11.2	5.8
	Multi-isocenter	2164.5	66.2	4.4	17.1	12.2	5.8
10	Single-isocenter	4633.7	61.3	3.8	18.7	10.8	4.4
	Multi-isocenter	4633.7	59.5	3.3	18.8	9.4	3.1
11	Single-isocenter	2770.3	55.1	6.1	28.7	19.1	8.4
	Multi-isocenter	2770.3	60.2	6.5	28.6	20.8	9.6

Table S2. Single-isocenter versus multi-isocenter plan dosimetric parameters for heart

Patient	Plan type	Volume (cm^3)	D_{max} (Gy)	D_{mean} (Gy)	V5 (%)	V10 (%)	V20 (%)
5	Single-isocenter	776.8	2.4	0.2	0	0	0
	Multi-isocenter	777.0	2.5	0.2	0	0	0
7	Single-isocenter	618.1	18.6	3.9	23.2	2.5	0
	Multi-isocenter	618.1	21.1	3.6	23.0	4.0	0.008
10	Single-isocenter	947.3	12.2	0.9	3.6	0.14	0
	Multi-isocenter	947.3	7.8	0.4	0.3	0	0
11	Single-isocenter	1161.9	14.9	2.0	10.8	0.7	0
	Multi-isocenter	1161.9	16.5	1.5	6.0	0.8	0

Table S3. Single-isocenter versus multi-isocenter plan dosimetric parameters for trachea

Patient	Plan type	Volume (cm^3)	D_{max} (Gy)	D_{mean} (Gy)	V5 (%)	V10 (%)	V20 (%)
5	Single-isocenter	21.1	13.5	4.6	44.5	14.7	0
	Multi-isocenter	21.1	18.5	4.3	40.6	11.7	0
7	Single-isocenter	23.6	22.1	2.8	8.6	5.5	0.3
	Multi-isocenter	23.7	24.7	1.9	10.6	4.8	0.6
10	Single-isocenter	19.2	23.6	7.7	71.2	18.3	3.8
	Multi-isocenter	19.2	18.9	6.6	63.5	15.6	0
11	Single-isocenter	11.9	10.4	5.2	44.0	0.2	0
	Multi-isocenter	11.9	13.5	6.5	68.4	10.0	0

Table S4. Single-isocenter versus multi-isocenter plan dosimetric parameters for esophagus

Patient	Plan type	Volume (cm^3)	D_{max} (Gy)	D_{mean} (Gy)	V5 (%)	V10 (%)	V20 (%)
5	Single-isocenter	21.1	40.3	14.7	30.5	0.4	0
	Multi-isocenter	21.1	12.0	2.5	24.7	2.1	0
7	Single-isocenter	104.4	21.1	7.7	76.3	21.0	0.06
	Multi-isocenter	104.4	15.0	4.2	32.5	0.2	0
10	Single-isocenter	15.6	17.4	4.4	38.3	14.8	0
	Multi-isocenter	15.6	6.8	2.7	21.3	0	0
11	Single-isocenter	49.8	5.4	2.2	0.5	0	0
	Multi-isocenter	49.8	6.8	2.5	13.8	0	0

Table S5. Single-isocenter versus multi-isocenter plan dosimetric parameters for spinal cord

Patient	Plan type	Volume (cm^3)	D_{max} (Gy)	D_{mean} (Gy)	V5 (%)	V10 (%)	V20 (%)
5	Single-isocenter	39.0	7.3	1.8	15.1	0	0
	Multi-isocenter	39.0	9.6	1.9	16.6	0	0
7	Single-isocenter	18.6	10.8	7.5	97.5	0.9	0
	Multi-isocenter	18.6	9.9	5.4	61.5	0	0
10	Single-isocenter	33.7	9.2	2.2	17.8	0	0
	Multi-isocenter	33.7	5.9	1.6	1.6	0	0
11	Single-isocenter	60.1	5.4	1.9	0.4	0	0
	Multi-isocenter	60.1	6.0	1.9	2.4	0	0

Table S6. Evaluation parameters for single-isocenter treatment plans

Patient	Combined PTV (cm^3)	Conformity Index	Homogeneity Index	Gradient Index	Gradient Distance (cm)
1	51	1.15	1.28	4.43	1.53
2	30.39	1.13	1.18	4.56	1.31
3	36.33	1.15	1.19	5.49	1.63
4	54.44	1.15	1.19	4.67	1.64
5	14.9	1.19	1.25	5.72	1.25
6	65.08	1.41	1.18	4.3	2.16
7	46	1.21	1.2	4.16	1.41
8	69.34	1.08	1.25	4.6	1.73
9	87.38	1.09	1.22	7.37	2.69
10	15.21	1.53	1.18	6.1	1.45
11	75.18	0.97	1.16	4.41	1.64
**Mean**	49.6	1.19	1.21	5.07	1.68
**Median**	51.0	1.15	1.19	4.60	1.63
**Std dev**	23.8	0.16	0.04	0.99	0.42
**Range**	14.9-87.4	0.97-1.53	1.16-1.28	4.16-7.37	1.25-2.69

Table S7. Evaluation parameters for multi-isocenter treatment plans

	**Target**	Combined PTV (cm^3)	Conformity Index	Homogeneity Index	Gradient Index	Gradient Distance (cm)
5	RUL, Lesion 1	5.6	1.22	0.94	6.98	1.14
	RUL, Lesion 2	9.3	1.25	1.17	6.49	0.97
7	RLL, Lesion 1	10.9	1.13	1.25	4.34	1.17
	RLL, Lesion 2	35.1	1.22	1.20	4.64	0.96
10	RLL, Lesion 1	7.3	1.14	1.16	5.34	0.93
	RUL, Lesion 2	8	1.14	1.15	5.60	0.99
11	LUL, Lesion 1	40.4	2.84	1.20	3.76	1.21
	LUL, Lesion 2	34.7	1.27	1.25	4.12	1.29
**Mean**	-	18.9	1.40	1.17	5.16	1.08
**Median**	-	10.1	1.22	1.19	4.99	1.07
**Std dev**	-	14.9	0.58	0.10	1.15	0.14
**Range**	-	5.6-40.4	1.13-2.84	0.94-1.25	3.76-6.98	0.93-1.29
